# Effect of Macular Internal Limiting Membrane Peeling on Single Surgery Success Rates of Vitrectomy for Uncomplicated, Primary Macula-Off Retinal Detachment

**DOI:** 10.1177/24741264231155352

**Published:** 2023-03-10

**Authors:** Rosanna K. Martens, Chao Chen, David S. Ehmann, Mark Greve, Mark E. Seamone

**Affiliations:** 1 Department of Ophthalmology and Visual Sciences, University of Alberta, Edmonton, AB, Canada

**Keywords:** epiretinal membrane, internal limiting membrane, retinal detachment, macular peeling, proliferative vitreoretinopathy, vitrectomy, recurrent retinal detachment, vitreoretinal surgery complications

## Abstract

**Purpose:** To determine the anatomic and visual outcomes of pars plana vitrectomy for uncomplicated, primary macula-off rhegmatogenous retinal detachment (RRD) with and without internal limiting membrane (ILM) peeling. **Methods:** This retrospective chart review comprised 129 patients with uncomplicated, primary macula-off RRD presenting between January 1, 2016, and May 31, 2021. Thirty-six patients (27.9%) had ILM peeling and 93 (72.0%) did not. The primary outcome was the rate of recurrent RRD. Secondary outcomes included preoperative and postoperative best-corrected visual acuity (BCVA), epiretinal membrane (ERM) formation, and macular thickness. **Results:** No significant difference was found in the risk for recurrent RRD between patients who had ILM peeling and those who did not (2.8% [1/36] and 5.4% [5/93], respectively) (*P* = 1.00). The final postoperative BCVA was better in eyes that did not have ILM peeling (*P<* .001). No ERM occurred in the group with ILM peeling, whereas ERM occurred in 27 patients (29.0%) who did not have ILM peeling. The temporal macular retina was thinner in eyes in which ILM peeling was performed. **Conclusions:** The risk for recurrent RRD was not statistically lower in eyes having ILM peeling of the macula in uncomplicated, primary macula-off RRD. Despite a reduction in postoperative ERM formation, eyes having macular ILM peeling had worse postoperative VA.

## Introduction

Peeling the internal limiting membrane (ILM) has become a commonly used technique in vitreoretinal surgery.^1^ It is performed for a variety of retinal conditions, including epiretinal membrane (ERM), full-thickness macular holes, macular edema, and vitreomacular traction.^1^ ERM is a common complication of rhegmatogenous retinal detachment (RRD) repair and occurs at an incidence of 6.1% to 35.7%.^2–7^ Macular ILM peeling has been used in RD repair because it is believed that removing the ILM prevents it from being a scaffold for cells to proliferate and create an ERM. It has been shown that ILM peeling in RD repair decreases the incidence of postoperative ERM formation.^5–12^

There is still debate on whether macular ILM peeling in RD leads to an improvement in visual acuity (VA). Although some studies found better visual outcomes after ILM peeling,^5,6,8^ others reported equivalent or worse visual outcomes.^7,9–11^ ILM peeling of a detached macula can be challenging. Peeling can cause inner retinal defects and retinal thinning.^10,13–16^ The risks of ILM peeling include inadvertent trauma, bleeding, scotoma, and toxicity of stains, such as indocyanine green (ICG). These factors can influence visual outcomes.

Finally, an important question is whether ILM peeling reduces the recurrence of RRD. It is thought that ILM peeling may reduce tractional forces transmitted to the macula from the periphery and vitreous base.^17^ In proliferative vitreoretinopathy (PVR), ILM peeling has been shown to be effective in reducing recurrence^17,18^; however, whether it is effective in primary RRD with no complicating factors remains under debate. One study^6^ found a reduced redetachment rate in eyes that had ILM peeling; however, the overall recurrence rate was high.

In this study, we assessed the effect of macular ILM peeling on the rate of anatomic reattachment in individuals with primary macula-off RRD in the absence of complicating factors. Secondary outcomes included the effect of macular ILM peeling on postoperative best-corrected VA (BCVA), postoperative macular thickness, and ERM formation

## Methods

This retrospective consecutive case series comprised patients who had vitrectomy for primary macula-off RD between January 1, 2016, and May 31, 2021. Surgery was performed at the same institution by 7 surgeons. All follow-up visits until December 18, 2021, were included. Institutional review board/ethics committee approval was obtained (#Pro00111001). Records were searched through the Healthquest electronic medical records database (Microquest, Inc). The records were filtered with the search terms “retinal detachment”, “C_3_F_8_”, “PPV”, or a combination.

Patients were included if they had a history of primary macula-off RD and were treated with a pars plana vitrectomy (PPV) with perfluoropropane (C_3_F_8_) gas as a tamponade. Patients were excluded if they had a history of tractional or exudative etiology, recurrent RRD, previous vitreoretinal surgery, a history of trauma or uveitis, cataract surgery complicated by posterior capsule rupture, a dislocated intraocular lens, scleral buckling (SB), preexisting ERM formation, or PVR.

Data collected included age, sex, axial length (AL), macular involvement, length of follow-up, preoperative BCVA, and method of subretinal fluid drainage as well as postoperative outcomes including BCVA, ERM formation, macular thickness, recurrence of RD, and surgical complications (hyphema, posterior capsule opacification [PCO], vitreous hemorrhage, choroidal effusion, high intraocular pressure, endophthalmitis). Time from RD to presentation was recorded when possible. ERMs were graded based on the grading scheme proposed by Govetto et al.^19^

It was not possible to reliably record the height of subretinal fluid on optical coherence tomography (OCT) before surgery. Retinal thickness was recorded using Early Treatment Diabetic Retinopathy Study zones on OCT with retina thickness maps (Heidelberg Spectralis OCT). With the OCT settings routinely used, the image size does not capture the superior outer and inferior outer macula in full; thus, these areas were excluded.

In all patients, 18% C_3_F_8_ gas was used as a tamponade. A previous study of tamponades used at our institution^20^ found that C_3_F_8_ had a higher success rate then sulfur hexafluoride (SF_6_) in RD repair. Therefore, SF_6_ is not often used at the institution, and cases in which it was used were excluded to prevent the effect of different tamponades on the results. Perfluoro-N-octane was not used in any case.

The decision to peel the ILM was made by the surgeon intraoperatively. A 23-gauge vitrectomy was performed with the Constellation Vision System (Alcon, Inc). The surgeon performed combined phacoemulsification and PPV when the patient did not have preexisting pseudophakia using a previously described technique.^19^ The ILM was stained with 0.3 cc of diluted ICG (5 mg/cc) under a balanced salt solution and rinsed after 15 seconds of application. Then, the ILM was removed out to the arcades with an ILM forceps (Greishaber Revolution DSM, Alcon, Inc). After air–fluid exchange, endolaser retinopexy to the retinal breaks was performed and C_3_F_8_ was injected.

The BCVA was measured before surgery and at each subsequent follow-up. The VA at the last follow-up visit was used as the final acuity. A neodymium:YAG capsulotomy was performed in eyes with visually significant PCO after surgery.

Statistical analyses were performed using SPSS software (version 28, IBM, Inc). The Mann-Whitney U test and Student *t* test were used to compare the differences between non-normally distributed and normally distributed continuous dependent variables, respectively, between independent groups. The Fisher exact test was used to compare the difference in categorical dependent variables. A *P* value of 0.05 was considered statistically significant.

## Results

### Demographics

The study included 129 patients. Of these patients, 36 (27.9%) had ILM peeling and 93 (72.0%) did not. All patients had a primary macula-off RRD that was repaired by PPV with C_3_F_8_. Table 1 shows the differences between eyes that had ILM peeling and those that did not. No patient had evidence of a chronic RD at presentation.

**Table 1.** Differences Between Eyes With and Eyes Without ILM Peeling During Retinal Detachment Repair.

Parameter	No ILM Peel (n = 93)^b^	ILM Peel (n = 36)^b^	*P* Value
Recurrence of RRD, n (%)	5 (5.38)	1 (2.78)	1.0
ERM formation, n (%)	27 (29.03)	0	<.001^c^
Mean BCVA			
Preoperative			
LogMAR	1.48 ± 0.70	1.44 ± 0.66	.974
Snellen	20/603	20/550	
Postoperative			
LogMAR	0.33 ± 0.32	0.47 ± 0.26	<.001^c^
Snellen	20/43	20/59	
Sex (n)^a^			
Male	65	28	.51
Female	28	8	
Mean age (y)	64.2 ± 8	64.3 ± 7	.94
Eye			
Right	40	16	
Left	53	20	1.0
Median onset of symptoms (d)	4	5	.128
Retinotomy rate, n (%)	14 (15.1)	5 (13.9)	.102
360 degrees laser, n (%)	4 (4.3)	4 (11.1)	.218
Mean AL (mm)	25.28 ± 1.99	24.75 ± 1.28	.138
Combined cataract/PPV, n (%)	53 (57.0)	18 (50.0)	.555
Mean follow-up (d)	387.54 ± 374.15	305.97 ± 237.89	.49

Abbreviations: AL, axial length; BCVA, best-corrected visual acuity; ERM, epiretinal membrane; ILM, internal limiting membrane; n, number of eyes; PPV, pars plana vitrectomy.

a Number of patients.

b Means ± SD.

c Statistically significant.

### Rate of Recurrent Retinal Detachment

**Figure 1.**
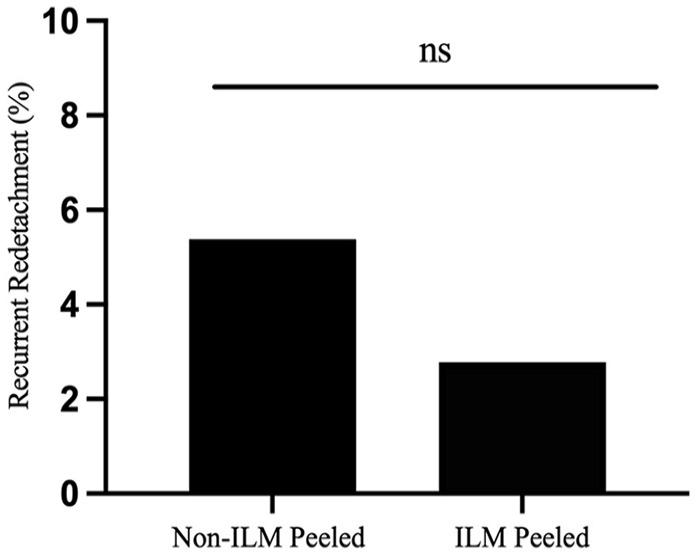
Percentage of recurrent retinal detachment postoperatively by group after primary retinal detachment repair with pars plana vitrectomy. Abbreviations: ILM, internal limiting membrane; ns, not statistically significant.

Recurrent RD occurred in 1 eye (2.78%) that had ILM peeling and in 5 eyes (5.38%) that did not (Figure 1). The difference was not statistically significant (*P* = 1.00). Therefore, despite theoretically relieving tractional forces from the macula, ILM peeling did not lead to a lower rate of recurrent RD.

### Postoperative Visual Acuity

**Figure 2.**
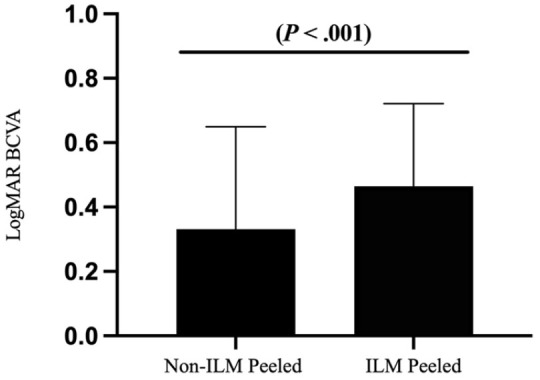
Final postoperative logMAR BCVA by group after primary retinal detachment repair with pars plana vitrectomy. Abbreviations: BCVA, best-corrected visual acuity; ILM, internal limiting membrane.

Table 1 shows the preoperative and postoperative BCVA by group. There was no statistically significant difference in the preoperative BCVA between patients who had ILM peeling and those who did not. There was, however, a statistically significant between-group difference in the postoperative BCVA, with better acuity in eyes that did not have ILM peeling (*P<* .001) (Figure 2).

### Rate of Epiretinal Membrane Formation

**Figure 3.**
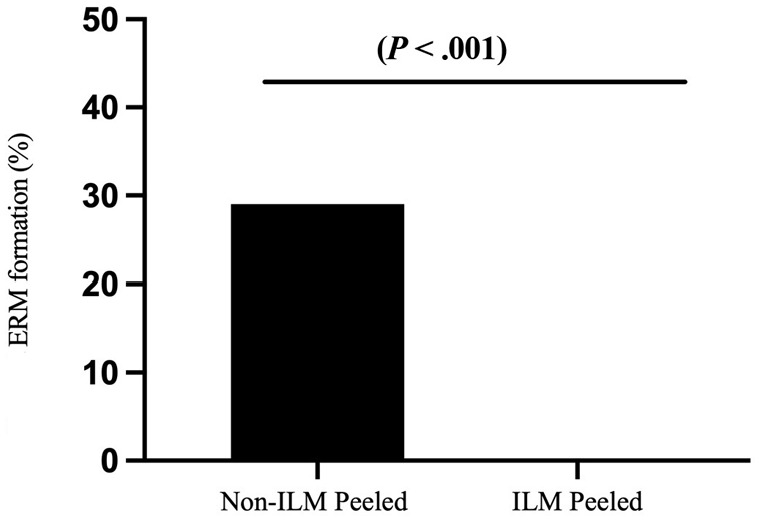
Percentage of ERM formation by group after primary retinal detachment repair with pars plana vitrectomy. Abbreviations: ERM, epiretinal membrane; ILM, internal limiting membrane.

At the final follow-up visit, an ERM developed in 27 patients (29.0%) who did not have ILM peeling; no patient who had ILM peeling developed an ERM (Figure 3). The difference between groups was statistically significant (*P<* .001). Although there was increased ERM formation in the group that did not have ILM peeling, it did not result in worse VA. Of the patients with ERM formation, 17 had grade 1, 6 had grade 2, 3 had grade 3, and 1 had grade 4. Four patients with ERM formation required secondary surgery for ERM removal. The indications for surgery were decreased vision or metamorphopsia with grade 3 or 4 ERM.

### Retinal Thickness

Table 2 shows the retinal thickness by groups. The inner temporal, outer temporal, inner superior, and inner inferior zones were statistically significantly thinner in eyes that had ILM peeling than in eyes that did not. The central subfield thickness and nasal retinal thickness were not statistically significantly different between the groups.

**Table 2.** Retinal Thickness With and Without ILM Peeling.^a^

Thickness	Mean ± SD (µm)		*P* Value
	No ILM Peel (n = 93)	ILM Peel (n = 36)	
Central subfield	302.56 ± 46.58	306.71 ± 40.48	.25
Inner superior	344.40 ± 22.89	319.00 ± 22.16	<.001^b^
Inner nasal	353.42 ± 33.37	348.57 ± 25.10	.49
Inner inferior	341.13 ± 24.77	325.86 ± 20.55	.005^b^
Inner temporal	333.70 ± 29.13	294.82 ± 22.42	<.001^b^
Outer nasal	325.11 ± 24.27	318.11 ± 17.73	.18
Outer temporal	287.65 ± 19.41	263.82 ± 15.73	<.001^b^

Abbreviation: ILM, internal limiting membrane.

a Retinal thickness was recorded using Early Treatment Diabetic Retinopathy Study zones on optical coherence tomography.

b Statistically significant.

## Conclusions

In this study, we compared the anatomic and the functional outcomes of 129 patients with primary macula-off RRD repaired using primary PPV and found that ILM peeling did not increase the rate of anatomic reattachment in these cases. In addition, although macular ILM peeling reduced the rate of ERM formation, this did not correspond with improved BCVA.

Previous studies have evaluated ILM peeling for RD repair^7,9,10,11,21^; many assessed peeling in complicated repair, such as PVR or SB with silicone oil.^5,6,11,17,18^ Our study is unique in that it specifically addressed primary macula-off detachments repaired using vitrectomy with C_3_F_8_ as a tamponade with no complicating risk factors, such as PVR, chronic/recurrent RD, or SB.

In cases of RRD complicated by PVR, ILM peeling has been shown to reduce redetachment rates.^17,18^ The hypothesis is that ILM peeling of the macula could result in reduced transmission of traction from the periphery and vitreous base and increased macular compliance.^17^ The impact on primary RRD without PVR is less well studied. In a small study,^6^ there was reduced redetachment; however, there was a high redetachment rate overall. In our study, which excluded complicating factors, we found no statistically significant difference in redetachment rates. This suggests that ILM peeling might not help prevent primary macula-off RRD in cases without PVR or other complicating factors.

The literature on the effect of ILM on final VA shows mixed results. Garweg et al^6^ found a reduction in ERMs as well as a positive effect on VA in eyes with macula-off RDs that had ILM peeling. A study by Forlini et al^5^ also found better VA outcomes; however, the study included macula-on RDs and macula-off RDs as well as complicating preoperative factors such as PVR, macular holes, and various surgical approaches, including SB and silicone oil. Other studies found no improvement in VA in eyes with ILM peeling despite a reduction in ERM formation.^7,9–12^

In our study, the postoperative BCVA was better in the group that did not have ILM peeling. There was no significant difference in age, sex, AL, preoperative VA, or the complication rate between eyes that did not have ILM peeling and eyes that did. Furthermore, the VA findings were not confounded by cataract because all patients were pseudophakic by the end of their primary RD repair given that cataract surgery is routinely performed at the time of vitrectomy for RD repair at our institution. Therefore, although it is possible that patients who had macular ILM peeling had worse RDs, our data support that our groups were equal. This suggests that patients could achieve better VA outcomes when the ILM is not peeled during vitrectomy for primary RD repair.

It is possible that the better BCVA in patients who did not have ILM peeling was related to secondary effects of ILM peeling. Possible complications of ILM peeling include a dissociated nerve fiber layer, retinal holes, damage to Müller cells, and stain toxicity.^14^ Furthermore, ILM peeling can also be more difficult when the retina is detached.

A study using OCT to detect structural changes in the retina^10^ found worse VA after ILM peeling and retinal dimples in that group. It also found reduced mean and foveal sensitivity measured by microperimetry. The retinal dimples are thought to be secondary to a dissociated optic nerve fiber layer, as first described by Tadayoni et al.^16^ Several other studies also reported inner retinal defects and thinning from ILM peeling.^7,15,21^

In our study, the temporal, superior, and inferior retina was thinner in eyes with ILM peeling. This finding is consistent with results in a study by Kumagai et al,^15^ who found temporal thinning in eyes after idiopathic ERM surgery and macular hole surgery. Outcomes could also be affected by staining with ICG, which is known to be cytotoxic, with possible longstanding effects on visual fields and optic nerve function.^22^

Consistent with many other studies,^5,6,8–11^ we found that ILM peeling during PPV for RD repair reduced ERM formation. The preventative benefit of ILM removal is thought to be from the removal of a scaffold for the ERM to grow on and also removal of the cellular components that could develop into an ERM.^23,24^ We found that peeling the ILM reduced the formation of ERM postoperatively, from 29.0% to 0%. Of cases that developed ERM, 85% (23/27) were grade 1 or 2 membranes. Therefore, although ERM is common after RD, the membranes are often low grade and unlikely to be visually significant.

Studies of surgery for secondary ERMs that develop after initial RD repair report good visual outcomes.^4,25,26^ A small percentage of patients who develop an ERM after RD require subsequent surgery. A study by Katira et al^3^ found that although 12.8% of eyes with primary RRDs repaired with PPV developed ERMs, only 4.3% required secondary surgery. In our study, the formation of ERM did not lead to a reduction in VA in eyes that did not have ILM peeling. Accordingly, peeling the macula to prevent ERM formation may not correlate with improve visual outcomes in all cases.

This study has limitations. It was not prospective or randomized, and there may have been some bias in determining which patients received ILM peeling. All our patients presented with macula-off RD; thus, preexisting ERM could have been missed because it would not be captured on OCT imaging. The surgeons at our institutions make a record of ERMs noted during surgery; therefore, clinically apparent ERMs would have been documented and excluded, although a subtle ERM could have been undetectable in the operating room. Another limitation is that some patients could have developed an ERM after their last follow-up and thus it would not have been captured. The high incidence of postoperative ERM in our study could be the result of our definition, which was the presence of any preretinal membrane in the macula on OCT; that membrane might not have been visually significant. Finally, because ERM can progress with time, longer follow-up is needed to determine whether the ERMs in our cases caused a significant effect in the long term.

In conclusion, ILM peeling of the macula in primary, uncomplicated macula-off RRD did not have a statistically significant benefit in reducing the risk for recurrent RRD (2.78% for ILM peeling vs 5.38% for no ILM peeling; *P* = 1.00). Despite a reduction in postoperative ERM formation (0% for ILM peeling vs 29.0% for no ILM peeling; *P<* .001), eyes that had macular ILM peeling had worse postoperative BCVA (20/59 for ILM peeling vs 20/43 for no ILM peeling; *P<* .001). These findings are of clinically significant because they may influence our surgical planning in the future for patients with primary macula-off RD.
